# Life on the rocks: unexpected enzyme activity of the extremophilic black fungus *Knufia chersonesos*

**DOI:** 10.3389/fbioe.2025.1720118

**Published:** 2026-01-12

**Authors:** Sophia Mihalyi, Christian Zimmermann, Felice Quartinello, Donatella Tesei, Astrid R. Mach-Aigner, Georg Guebitz, Doris Ribitsch

**Affiliations:** 1 Institute of Environmental Biotechnology, Department of Agricultural Sciences, IFA Tulln, BOKU University, Tulln an der Donau, Austria; 2 Institute of Chemical, Environmental and Bioscience Engineering, Faculty of Technical Chemistry, TU Wien, Vienna, Austria; 3 Institute of Microbiology and Microbial Biotechnology, BOKU University, Vienna, Austria

**Keywords:** cutinase, lipase, PBAT hydrolysis, *Trichoderma* expression, adipic acid, black fungi

## Abstract

Knowledge about extremophile organisms and their survival strategies could be of great value for various industrial applications including biological plastics recycling. The black fungus *Knufia chersonesos* inhabits extreme environments such as rocks and therefore produces specific enzymes that function under harsh conditions. A cutinase (Kc_Cut) and a lipase (Kc_Lip) identified *via* proteomics-based screening in the secretome of *K. chersonesos* grown on poly (butylene-adipate-*co*-terephthalate) (PBAT) as carbon sources were recombinantly expressed in *Komagataella phaffii* and *Trichoderma reesei*, respectively, for further characterization. The purified enzymes showed a specific activity of 83 ± 1 and 0.23 ± 0.02 U mg^-1^ on *para*-nitrophenylbutyrate (*p*-NPB) as substrate, respectively. Optimum conditions of Kc_Cut were evaluated through activity measurements on *p*-NPB and resulted in 50 °C, pH 8 and 100 mM potassium phosphate buffer. Incubation with PBAT powder for 72 h resulted in the release of 17 ± 1 µM of terephthalic acid (Ta) by Kc_Cut while the Kc_Lip liberated the trimer BTaB. This indicated a cooperative action of the two enzymes which was confirmed by hydrolysis of BTaB by Kc_Cut and provides valuable insight into the metabolic potential and adaptability of *K. chersonesos*.

## 1 Introduction

Microcolonial fungi (MCF) and black yeasts, grouped under the general name black fungi, are a group of extremotolerant fungi which are particularly adapted to survival in extreme and nutrient-poor environments such as bare rocks, deserts, and even outer space (Marzban et al., 2013). Besides, species from plants, insects, and clinical samples have been additionally reported (Gostinčar et al., 2022). Although black fungi represent a heterogeneous taxonomic group, they share a set of traits, the most prominent being melanin pigmentation. These organisms produce melanin within their thick cell walls, which absorbs or deflects UV radiation, thereby reducing damage caused by the sun and even making them tolerant to toxic metals, providing ecological advantages in polluted or highly radioactive sites (Catanzaro et al., 2024). Moreover, MCF are among the first life forms to colonize bare rock in extreme environments. They inhabit rock surfaces, pores and fissures–often created by their own deteriorative activity–where they break down the substrate through physical and chemical processes, this contributing to the formation of soil over long periods.

An important representative of black fungi is the genus *Knufia* within the family *Trichomeriaceae*, order *Chaetothyriales*, and phylum Ascomycota (Chander et al., 2024). The genus *Knufia* currently comprises 15 known species, including the well-established species *Knufia cryptophialidica* (Hutchison et al., 1995), *Knufia epidermidis* (Li et al., 2008), and *Knufia peltigerae* (Tsuneda et al., 2011). These species typically occur in various habitats, including soils, algae, and hydrocarbon-contaminated sites, with varying psychrophilic (cold-loving) and salt-tolerant characteristics. *Knufia chersonesos* (synonym *Knufia petricola*) is a melanized, non-sporulating, stress-tolerant rock-inhabiting fungus first isolated from Crimean marble (Tsuneda et al., 2011) and now reported from the Mediterranean to the Arctic (Wollenzien et al., 1997). Its resilience includes thermo- and desiccation tolerance and survival at ozone levels far exceeding those harmful to plants and animals. Nutritional studies further showed growth on monoaromatic compounds (Nai et al., 2013). Recently, it has been demonstrated that *K. chersonesos* can even grow in presence of the synthetic copolyester polybutylene adipate terephthalate (PBAT) as additional or sole carbon source (Tesei et al., 2020). PBAT is a biodegradable aliphatic-aromatic copolyester that has attracted considerable attention as an environmentally friendly alternative to plastic and is therefore increasingly being used in agriculture, for example, (Roy et al., 2024). The polymer combines flexible aliphatic segments (butylene adipate) with rigid aromatic segments (butylene terephthalate), offering a balanced ratio between flexibility and mechanical strength. PBAT can be degraded by microorganisms under composting conditions, in soil, and in marine environments (Fernandes et al., 2024; Nakayama et al., 2019). Examples for other reported PBAT-hydrolyzing fungi include *A. alternata* (Fei et al., 2024), *P. lilacinum* (Tseng et al., 2023), *Humicola* and *Schizothecium* (Wang et al., 2025) where hydrolysis took at least 30 days for *Alternaria alternata* and *Purpureocillium lilacinum* up to 180 days in soil communities.

In nature, the biodegradation of synthetic polymers relies on microorganisms that colonize surfaces and secrete extracellular enzymes to depolymerize polymers into low-molecular-weight compounds that can then be taken up and metabolized by the cell (Zumstein et al., 2018). This extracellular depolymerization is often carried out by cutinases, which naturally hydrolyze the polyester cutin from plants, but also cleave other aliphatic and aromatic polyesters and occur in bacteria and fungi. A well-known enzyme for hydrolysis of various polyesters including PBAT is a cutinase from *Humicola insolens* (HiC) but also recently discovered and optimized enzymes such as a new hydrolase from *Thermoanaerobacterales* (Thb) have demonstrated such activities (Carniel et al., 2017; Siracusa et al., 2025). Further known hydrolases that efficiently hydrolyze the commonly applied PBAT include, e.g., a leaf-branch compost cutinase (LCC) (Ismail et al., 2024; Shirke et al., 2018) and esterases from *Clostridium botulinum* (Perz et al., 2016) as well as PETase from *Ideonella sakaiensis* that shows structural features common in cutinases and lipases (Austin et al., 2018) and it`s homolog Rhb from *Rhizobacter* sp. (Chalwatzis et al., 2025). Very recently, cutinases from *Gordonia* species have proven activity on PBAT (Steiner et al., 2025). However, the specific enzymes that catalyze the activity on the polyester PBAT in *K. chersonesos* have not yet been characterized. Nevertheless, sequences of potential polyester hydrolases were identified using shotgun proteomics of cultures whose enzyme activities were induced by addition of PBAT polyester during cell growth (Tesei et al., 2020).

In this study, we heterologously expressed two putative PBAT hydrolysing enzymes from *K. chersonesos*, a secretory lipase Kc_Lip in *Trichoderma reesei* and an extracellular cutinase Kc_Cut in *Komagataella phaffii* and demonstrated their contribution in PBAT depolymerization. The aim of this research is to gain more insights into the substrate converting mechanism and potential of the extremophilic organism *K. chersonesos.*

## 2 Materials and methods

### 2.1 Chemicals and polymers

#### 2.1.1 Cultivation media

YPD cultivation media consisted of 10 g L^-1^ yeast extract, 20 g L^-1^ peptone, 20 g L^-1^ glucose, and 100 μg mL^-1^ Zeocin and/or 15 g L^-1^ Agar if needed. BM(M)Y (Buffered Methanol-complex) medium consisted of 10 g L^-1^ yeast extract, 20 g L^-1^ peptone, 100 mM phosphate buffer pH 6, 1.34% YNB (Yeast Nitrogen Base with Ammonium Sulfate without amino acids) and 4 × 10^−5^% biotin.

#### 2.1.2 Microorganisms

*Komagataella phaffii* KM71H was used for expression of the *K. chersonesos* cutinase (Kc_Cut). The *T. reesei* strains QM6a Δmus53 (Steiger et al., 2011), QM6a Δpyr4 (Derntl et al., 2015) and the herein constructed strain Lip_ex_ was used for expression of Kc_Lip. All *T. reesei* strains were maintained on malt extract (MEX) plates (3% malt extract, 0.1% peptone, 1.5% agar). 5 mM uridine was added if required. For liquid cultivations, 10^9^ spores L^-1^ were inoculated in YPD and incubated at 30 °C in an orbital shaker at 180 rpm for 72 h.

### 2.2 Gene design and heterologous expression

#### 2.2.1 Expression of the *K. chersonesos* cutinase

Kc_Cut was expressed recombinantly in *K. phaffii* KM71H. For this purpose, the gene encoding the cutinase with the HMMER accession number PF01083.21 was optimized for the *K. phaffii* codon usage, shortened by the nucleotide sequence of the natural signal peptide and C-terminally fused to a StrepTag II. The gene was cloned into the vector pPICZalphaB *via* the restriction sites XhoI and NotI by commercial service (GenScript Biotech, Leiden, Netherlands). The plasmid was linearized with SacI (New England Biolabs, Ipswich, MA, United States) and transformed into *K. phaffii* KM71H (ThermoFisher Scientific, Waltham, MA, United States) by electroporation according to manufacturer’s protocol (MikroPulserTM, Bio-Rad, Hercules, CA, United States). Transformed cells were plated on YPD-Zeocin 100 agar for 3 days at 28 °C. From several colonies, the gene of interest was analyzed through colony PCR. Afterwards, selected clones were again allowed to grow on agar plates and then cultivated as preculture in 1 L baffled flasks in YPD media at 28 °C, 250 rpm for 16–18 h until an OD_600_ of two to six was reached. For the main culture, cells were harvested by centrifugation at 3,000 *g* for 5 min and resuspended in 1/10th of the original volume with BM(M)Y medium and transferred into 100 mL baffled shake flasks. 100% methanol was added to a final concentration of 0.5% twice per day manually to induce the expression phase. After 72 h, the supernatant which contained the protein of interest was separated from the cells through centrifugation (5920 R, ThermoScientific, Austria). Expression levels were estimated through SDS-PAGE analysis for preparation of cryo-stocks of selected clones (Supplementary Figure S1). Cryo stocks were prepared from an overnight culture in a final concentration of 15% glycerol and frozen at −80 °C.

#### 2.2.2 Expression of the *K. chersonesos* lipase

Expression of Kc_Lip was performed in *T. reesei.* To enable expression and secretion of Kc_Lip, the secretion signal peptide from *T. reesei* CBHI (MYRKLAVISAFLATARAQ) was added to the N-terminus and the Strep-Tag II sequence (WSHPQFEK) to the C-terminus. The amino acids sequence was reverse translated with OPTIMIZER (Puigbò et al., 2007) using the guided random method and the codon usage table for *Hypocrea jecorina* (teleomorph of *T. reesei* (Kuhls et al., 1996) published on Codon Usage Database (https://www.kazusa.or.jp/codon/). The sequence was further manually curated to remove recognition sequences for the endonucleases used for the following cloning purposes. Additionally, the recognition sites for AflII and SpeI were added to the 5′ and the 3′ end, respectively. Protein and DNA sequences are provided in Supplementary Material (Supplementary Figure S2). The gene was optimized for *T. reesei* and synthesized at GenScript Biotech (Leiden, Netherlands) and recognition sites for AflII and SpeI added. The synthetic Kc_Lip gene was cloned into pRP4-TX(WT) (Derntl et al., 2019) using AflII and SpeI (both NEB, Ipswich, MA, United States), putting the Kc_Lip gene under the control of the constitutive *tef1* promoter. The plasmid further contained the *pyr4* gene and suitable flanking regions allowing a targeted insertion of the lipase expression cassette at the *pyr4* locus (Supplementary Figure S3) as described in (Derntl et al., 2015).

The lipase-insertion plasmid was digested with NotI (NEB) and transformed into *T. reesei* QM6a Δpyr4 (Derntl et al., 2015) using a PEG-mediated transformation of protoplasts. Spores of the recipient strain were plated on sterile cellophane sheets laid on malt extract plates containing 5 mM uridine at 30 °C overnight. The mycelium was scraped off and transferred into 15 mL Buffer A (1.2 M sorbitol, 100 mM KH_2_PO_4_, pH 5.6) containing 600 mg Vinotaste Pro (Novonesis, Bagsværd, Denmark) and 0.5 mg chitinase from *Streptomyces griseus* (Sigma-Aldrich C6137). This mixture was incubated in a sterile Petri dish in an orbital shaker at 60 rpm and 30 °C for approx. 2–3 h until the mycelium was completely disintegrated. The suspension was filtered through a 70 µm cell sieve and incubated on ice for 5 min. The suspension was filled up to 40 mL with ice-cold 1.2 M sorbitol and centrifuged at 2,500 *g* at 4 °C for 10 min. The pellet was resuspended in 30 mL 1.2 M sorbitol and again centrifuged. The protoplasts were finally resuspended in 1 mL ice-cold Buffer B (1 M sorbitol, 25 mM CaCl_2_, 10 mM Tris.Cl, pH 7.5). Next, 20 µg of the linearized plasmid were filled up to 150 µL with ice-cold Buffer B, carefully mixed with 100 µL of the protoplast suspension and 100 µL 20% PEG (mixture of 6.7 mL Buffer B and 3.3 mL 60% PEG (60 g PEG 4000, 1 mL 1 M Tris-HCl pH 7.5, 1 mL 1 M CaCl_2_, 38 mL ddH_2_O)) in a 50 mL reaction tube. This mixture was incubated on ice for 30 min before 60% PEG was added in steps (50 μL, 200 μL, 500 µL). Next, the tube was incubated at room temperature for 20 min, and finally, Buffer C (1 M sorbitol, 10 mM Tris.Cl, pH 7.5) was added in steps (200 μL, 400 μL, 1 mL, 2.5 mL). For plating, the tube was filled up to 50 mL with molten, 50 °C-warm selection medium (MA medium (Mandels and Andreotti, 1978) lacking peptone containing 1% (w/v) glucose and 1 M sucrose) and poured into a 14.5 cm Petri dish. The plates were incubated at 30 °C under light until colonies were visible (up to a week). The candidates were then homokaryon selected by spore streaking on selection plates (without sucrose).

For genotyping, the mycelium from a liquid cultivation of *K. chersonesos* was harvested and pressed dry between two sheets of filter paper. Approx. 50 mg were lysed in 1 mL CTAB buffer (1.4 M NaCl, 100 mM Tris-HCl pH 8.0, 10 mM EDTA, 2% CTAB, 1% polyvinylpyrrolidone) with 0.37 g small glass beads, 0.25 g medium glass beads and one large glass bead in a 2 mL screw cap reaction tube using a Fast-Prep-24 (MP Biomedicals, Santa Ana, CA, United States) at 6 m/s for 30 s. The samples were incubated at 65 °C for 20 min and finally centrifuged at 12,000 *g* for 10 min. The supernatant was transferred to a 2 mL reaction tube, and the DNA was purified by a phenol-chloroform-isoamyl alcohol extraction, followed by two chloroform extractions. The samples were then treated with RNAse A (Thermo Fisher Scientific) according to the manufacturer’s instructions and the DNA finally precipitated using isopropanol and dissolved in 10 mM Tris-HCl pH 8.0. All PCR reactions for genotyping were performed with OneTaq DNA Polymerase (NEB) according to the manufacturer’s instructions. Additionally, we amplified the inserted synthetic gene using the primers tef1a_core_f–cbh2-rv and the Q5 polymerase (NEB), cloned it into pJET1.2 using the CloneJET PCR Cloning Kit (Thermo Fisher Scientific) according to the manufacturer’s instructions and confirmed the sequence by Sanger sequencing at Microsynth GmbH, Baldach, Switzerland (Supplementary Table S1).

### 2.3 SDS-PAGE and western blot analysis

To confirm the presence of the expressed enzyme, SDS-PAGE was performed as a first step. To detect the size of the protein without interference of possible glycosylation, a de-glycosylation step was applied beforehand with a modified protocol provided by New England Biolabs. Therefore, 10 µL of the protein of interest in glycoprotein denaturing buffer was heated to 100 °C for 10 min and afterwards incubated with Endo Hf in G5 reaction buffer at 37 °C for 1 h. Then Laemmli Buffer was added, the sample boiled for 10 min at 100 °C and proteins analyzed on SDS-PAGE (Mini-PROTEAN TGX precast stain-free, BioRad, United States).

After the cultivation of *T. reesei*, the mycelium was separated from the supernatant by filtration through miracloth, followed by a centrifugation at 9,000 g at 4 °C for 10 min. Finally, the supernatant was filtered through a 0.22 µm Steritop vacuum bottle top filter (MilliporeSigma, Burlington, MA, United States). 100 μL of the supernatant were mixed with 100 µL of 2x Laemmli Buffer (120 mM Tris, 4% (w/v) SDS, 17.4% (w/v) glycerol, 2% (v/v) 2-mercapthoethanol, 0.02% (w/v) bromophenol blue, pH 6.8 (HCl)) and incubated at 95 °C for 5 min 10 μL of the samples were loaded onto a discontinuous electrophoretic gel as described by Laemmli in 1970 (Laemmli, 1970). The gel electrophoresis was run in a Mini-PROTEAN Tetra Vertical Electrophoresis Cell (Biorad, Hercules, CA, United States) at 15 mA per gel until the bromphenol blue reached the end of the gel. The PageRuler Plus Stained protein ladder (Thermo Scientific) was used as an internal standard.

For a subsequent Western blot analysis, the gel was washed in distilled water for 5–10 min for three times and soaked in the cathode buffer (25 mM Tris, 40 mM 6-aminocaproic acid, 20% (v/v) methanol, pH 9.4 (HCl)) for 1–2 min directly before assembling the blot. The blot was assembled on a semi-dry blotter consisting of 3 layers of Whatman filter paper soaked in anode buffer II (300 mM Tris, 20%(v/v) methanol, pH 10.4 (HCl)), 3 layers of Whatman filter paper soaked in anode buffer I (25 mM Tris, 20% (v/v) methanol, pH 10.4 (HCl)), a piece of PVDF transfer membrane (Immobilon-P, 0.45 µm pore size, MilliporeSigma) which was previously activated in methanol and soaked in anode buffer I, the gel, and finally, 3 layers of Whatman filter paper soaked in cathode buffer. The proteins were transferred by applying an electric current (100 mA per blot, limited to 12 V) for 20 min. After disassembling the blot, the membrane was soaked in methanol and dried at ambient temperature to reduce the background signals. Next, the membrane was re-activated in methanol and equilibrated in 1x PBS (0.137 M NaCl, 2.7 mM KCl, 10 mM Na_2_HPO_4_, 1,76 mM KH_2_PO_4_, pH 7.4) for 5–10 min. Then, the membrane was blocked with 1x PBS containing 2% (w/v) bovine serum albumin (BSA) for 1 h and then washed twice with 1x PBS-T (PBS with additional 0.1% (v/v) Tween-20) for 5–10 min. The first antibody (polyclonal Strep epitope Tag antibody from rabbit, product GTX128061 GeneTex) was diluted 1:5,000 in 10 mL 1x PBS-T containing 0.3% (w/v) BSA and the membrane incubated in it for 1 h. The blot was washed 3 times in 1x PBS-T and incubated in 10 mL 1xPBS-T containing a goat anti rabbit antibody, conjugated with horse radish peroxidase, product 32,260 from Invitrogen). Finally, the blot was washed with 1x PBS three times. For visualization, the SuperSignal West Pico chemiluminescence substrate (Thermo Scientific) was used according to the manufacturer’s instructions and a ChemiDoc MP Imaging system (Biorad).

### 2.4 Enzyme purification

Purification of both enzymes from the cultivation supernatant was performed on an ÄKTA pure™ protein purification system (GE Healthcare, Chalfont St. Giles, UK) with a streptag column (IBA StrepTactin-XT-superflow 5 mL). First, the cultivation media was exchanged to the washing buffer (100 mM Tris-HCl pH 8, 150 mM NaCl) through a PD-10 desalting column (GE Healthcare Cytiva, United States) and sterile filtered through a 0.2 µm PES filter membrane. After elution (elution buffer contained additional 50 mM biotin), an SDS-PAGE analysis was performed to identify the fractions of interest that contained the enzyme. The selected fractions were pooled, concentrated and rebuffered into storage buffer (100 mM Tris-HCl pH 7) (exemplary image of an SDS-PAGE, Supplementary Figure S4). The protein concentration was measured on a nanophotometer (Implen, Germany). The concentration of Kc_Lip in the supernatant was increased 50x by membrane filtration with a 30 kDa MWCO (Vivaspin® 15R, Sartorius, UK) before purification. The purification process enabled an increase in specific activity from 52.1 ± 7.7 to 106.0 ± 2.5 [U mg^-1^] of Kc_Cut in the cultivation supernatant and after downstream processing, respectively.

### 2.5 Biochemical characterization

#### 2.5.1 *p-*NPB esterase activity assay

*Para-*Nitrophenylbutyrate (*p-*NPB) is enzymatically hydrolyzed to *p-*nitrophenol which shows a change in absorbance and can be detected at 405 nm. A substrate concentration of 15.75 mM was applied in the reaction and absorbance was measured for 10 min in a 96-well plate with a cycle of every 18 s on an Infinite 200 Pro spectrophotometer (Tecan, Switzerland). The activity was calculated through Equation 1 with V_f_ final volume (220 µL), V_e_ enzyme volume (20 µL), D_f_ dilution factor, d height of the solution (0.611 cm) and ε extinction coefficient (described below).

$$Volumetric\;activity\;\left[\frac{U}{mL}\right]=\frac{slope*V_{f}}{\varepsilon *d*V_{e}}*D_{f}$$ (1)

To calculate the volumetric activity from the *p-*NPB assay, the molar extinction coefficient was determined for each buffer in a concentration range from 0.01–0.2 mM at 405 nm. The slope of the calibration curve gives the molar extinction coefficient (Supplementary Table S2).

The temperature stability was investigated through incubation of the enzyme in Eppendorf tubes at different temperatures for 1 week and measurement of the activity every day at an enzyme concentration of 1.16 mg mL^-1^ (50 µM).

#### 2.5.2 Design of experiments (DoE)

The optimal conditions for hydrolysis of *p*-NPB by the Kc_Cut were evaluated through a full factorial design of experiments (DoE) planned with the MODDE Pro 13 (Sartorius) software. The chosen independent variables were pH (6, 7, 8), ionic strength (100, 500, 1,000 mM) and temperature (50, 60, 70 °C) for two different buffers (potassium phosphate and Tris-HCl). The response variable was the enzymatic activity measured through the *p-*NPB esterase activity assay following a random sequence. The scheme of experiments can be found in Supplementary Tables S3, S4. The MODDE output parameters resulted in R^2^ = 0.59, Q^2^ = 0.34, and model validity of 0.99 which represents a significant model.

### 2.6 Hydrolysis of PBAT

The hydrolysis of PBAT film and powder was performed in 2 mL Eppendorf tubes at the chosen optimum conditions. Between 9.5 and 10.5 mg of powder and 0.5 × 1 cm films were added to the reaction for 72 h. For all reactions, an enzyme concentration of 5 µM was applied.

#### 2.6.1 Detection of hydrolysis products

Solubilized hydrolysis products were quantified through high-performance liquid chromatography (HPLC) on an Agilent Technologies 1,260 Infinity instrument (Agilent Technologies, Santa Clara, CA, United States) with a C18 reversed-phase column connected to an UV detector. The mobile phase consisted of a gradient of methanol and 0.1% formic acid with a flow rate of 0.85 mL min^-1^. Calibration was performed with terephthalic acid from 0.001 mM–1 mM. Sample preparation included protein precipitation with ice-cold methanol (1:2), acidification with 6 N HCl to pH 3.5, centrifugation at 12,700 rpm (Eppendorf Centrifuge 5427 R) at 4 °C for 15 min and filtration through 0.45 μm PA filter.

## 3 Results and discussion

In a secretome screening of the black fungus *K. chersonesos* and its non-melanized mutant cultivated in the presence of PBAT, using qualitative and quantitative proteomics, we recently have identified enzymes that may play a role in the hydrolysis of PBAT (Tesei et al., 2020). In this study, these enzymes, namely, a cutinase termed Kc_Cut and a lipase termed Kc_Lip were recombinantly expressed and characterized related to PBAT hydrolysis.

### 3.1 Sequence analysis

Proteomics-based screening of *K. chersonesos* revealed that a secretory lipase (Kc_Lip, UniProtKb g1109.t1/A0A2K3Q6V2_9HYPO) was upregulated three-fold in the PBAT-exposed secretome of the wild-type strain, while an extracellular cutinase (Kc_Cut, UniProtKb g4295.t1/W2RQJ3_9EURO) was detected exclusively in the secretome of the mutant when cultured in the presence of PBAT. Sequence analysis showed that both enzymes have a signal peptide, indicating an extracellular occurrence in the liquid culture. Functional analysis using InterPro (Blum et al., 2024) revealed that Kc_Lip is a secreted lipase consisting of a typical α/β-hydrolase fold and involved in lipid metabolism in *Knufia*. Kc_Cut, on the other hand, is classified as a cutinase that is probably involved in carbohydrate catabolic process (Tesei et al., 2020).

**FIGURE 1:**
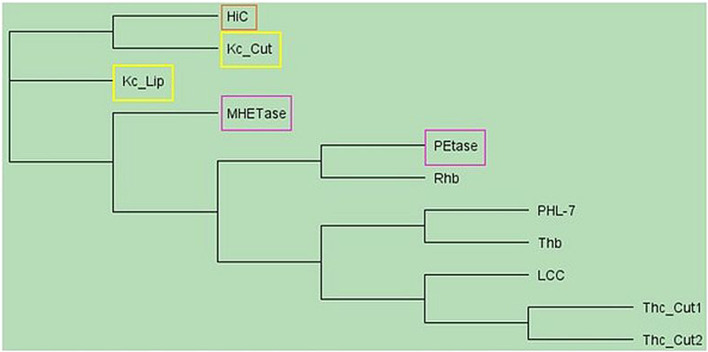
Phylogenetic tree of polyester hydrolyzing enzymes based on protein sequences without signal peptides. The phylogenetic tree was constructed by Geneious software employing the Neighbor-joining Method (Tamura et al., 2021) based on the protein sequences of enzymes used in this study. The protein sequences are named by their Cryptonym. The displayed enzymes and their accession numbers are Kc_Cut (PF01083.21), Kc_Lip (PF03583.13), PETase (WP_054022242), MHETase (A0A0K8P8E7), Thb (MBO2503201) LCC (G9BY57.1), PHL-7 (7NEI_A), Rhb (MBX3625601), HiC (AAE13316), Thc_Cut1 (ADV92526.1) and Thc_Cut2 (ADV92527.1). Yellow: *K. chersonesos* enzymes characterized in this study. Orange: highest homologous cutinase HiC. Pink: *Ideonella sarkaniensis* polyester hydrolases. References are provided in the text.

Alignment of sequences and calculation of the similarities using BLOSUM62 (Henikoff and Henikoff, 1992) revealed that Kc_Cut and Kc_Lip are only 5% similar to each other. Compared to known enzymes hydrolyzing PBAT, the highest homology (49%) of Kc_Cut was found to be with cutinase from *H. insolens* (Kold et al., 2014), whereas only low similarities were found with all other polyester hydrolases. Kc_Cut had similarity of only 15%–17% with LCC (Sulaiman et al., 2012), cutinases from *Thermobifida cellulosilytica* (Herrero Acero et al., 2011), Thb (Siracusa et al., 2025), PHL-7 (Sonnendecker et al., 2022) and PETase (Austin et al., 2018; Palm et al., 2019; Yoshida et al., 2016). In contrast, Kc_Lip showed only 10% and 14% similarity to HiC and MHETase, respectively. All other similarities were below 10%, which is probably also because Kc_Cut and Kc_Lip are of fungal origin, whereas known polyester hydrolyzing enzymes are predominantly of bacterial origin (Figure 1).

*Knufia* is a fungal genus, therefore proteins can be post-translationally modified by glycosylation to maintain their function and destination in the cell. In fact, the Kc_Lip amino acid sequence contains 10 potential N-glycosylation sites as predicted by the NetNGly server 1.0 (Gupta and Brunak, 2001), and Kc_Cut at least 3 Asn-Xaa-Ser/Thr sequons.

### 3.2 Gene design and heterologous expression

Due to their fungal origin, in a first step the eukaryotic expression host *K. phaffii* was selected for recombinant production of the enzymes from *K. chersonesos*. For this purpose, the genes for both enzymes were first codon optimized for *Komagataella* and cloned without their natural signal sequence into the vector pPICZalphaB, which enables the secretion of the enzymes from the cell by the α-factor. SDS-PAGE analysis of the culture supernatants after de-glycosylation with Endo H_f_ confirmed the expression of Kc_Cut by the presence of a protein band at the expected molecular weight of 22.36 kDa (Supplementary Figure S1), whereas no band at 52.51 kDa was detected for Kc_Lip (data not shown). Two transformants of Kc_Cut, colony 4 and colony 12 in Supplementary Figure S1, were selected and further investigated. Kc_Cut was purified by affinity chromatography from the culture supernatant and used for further biochemical characterization studies (Supplementary Figure S4). The activity assay using *p-*NPB as substrate showed a specific activity of 79 ± 5 U mg^-1^ for colony 4 and 83 ± 1 U mg^-1^ for colony 12.

Since expression of Kc_Lip in *K. phaffii* was unsuccessful, *T. reesei* was selected as expression host, as this genus has a different glycosylation pattern than *Komagataella* (Stals et al., 2004) and could therefore favor enzyme expression. *T. reesei* can express and secrete esterases in a natural state as well and hence, the cultivation supernatant from the wildtype was tested as reference (Supplementary Figure S5). Measuring the activity with the *p-*NPB esterase activity assay resulted in 33 ± 0 U L^-1^ from the wildtype and 53 ± 1 U L^-1^ in the transformed strain. This indicated expression of the lipase in the heterologous strain and upon concentration, the activity showed 182 ± 24 U L^-1^ and 576 ± 39 U L^-1^, respectively. Characterization of the enzyme solution after purification resulted in an activity of 137 ± 13 U L^-1^ with *p-*NPB and a protein concentration of 0.59 g L^-1^.

Both enzymes were successfully expressed as soluble and functional proteins in the selected hosts. However, expression performance could be further improved by optimizing several parameters. Codon optimization, for instance, is not universally beneficial—rare codons can create intentional “pause sites” that enable proper co-translational folding, and their removal may cause misfolding and aggregation. Therefore, different codon optimization algorithms could be tested to maximize soluble protein yields. Additionally, alternative signal peptides merit investigation, as they significantly influence expression levels, protein localization, secretion efficiency, and product quality. Empirical testing of these parameters could enhance both yield and protein quality.

### 3.3 Biochemical characterization

**FIGURE 2:**
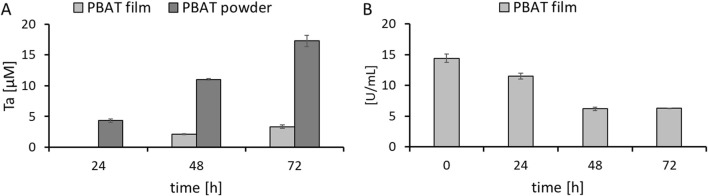
Contour response of *p*-NPB Kc_Cut activity at varying pH, temperature, and ionic strength in **(A)** potassium phosphate buffer and **(B)** Tris-HCl buffer.

To evaluate the optimum conditions for polymer hydrolysis, a design of experiments (DoE) was set up with the MODDE Pro 13 (Sartorius) software to evaluate different buffers, pH, and temperatures. Since the catalytic activity and stability of enzymes can change with the type of buffer, ionic strength and pH, two different commonly applied buffers (Tris-HCl and potassium phosphate) were tested in various concentrations and pH (Schmidt et al., 2016). Previously, highest activity for the isolated *K. chersonesos* supernatant was found at temperatures around 50 °C while cutinases generally prefer alkaline pH (Jiddah Usman et al., 2023; Tesei et al., 2020). Here, the response for the parameters was measured by activity on *p-*NPB and resulted in a contour graph that identified the preferred condition for the two tested buffers (Figure 2).

From the DoE, the preferred conditions for Kc_Cut were identified as 100 mM, pH 8 and 50 °C for potassium phosphate (KPO) and 1 M, pH 7 and 50 °C for Tris-HCl buffer. The activity was overall higher in KPO over Tris-HCl. Then, stabilizing salts and temperature stability were evaluated to ensure maximum activity and performance during enzymatic polyester hydrolysis.

Addition of 2 mM CaCl_2_ and 2 mM MgCl_2_ (Jiddah Usman et al., 2023) did not enhance enzyme activity and were therefore not included in further experiments (Supplementary Figure S6). The temperature stability measurement in the selected buffer for 7 days showed higher stability at a lower temperature of 40 °C. There was still more than 50% activity detected at 50 °C after 72 h. At 60 °C and 70 °C, the activity dropped below 50% after 3 h which does not present higher temperatures as suitable with this enzyme (Supplementary Figure S7). Kc_Lip did not show activity at 40 °C and 50 °C after 72 h. However, *K. chersonesos* is an extremophile organism in terms of nutrient-poor environments, which does not necessarily mean that it is also extremophile in terms of temperature.

### 3.4 Hydrolysis of PBAT

**FIGURE 3:**
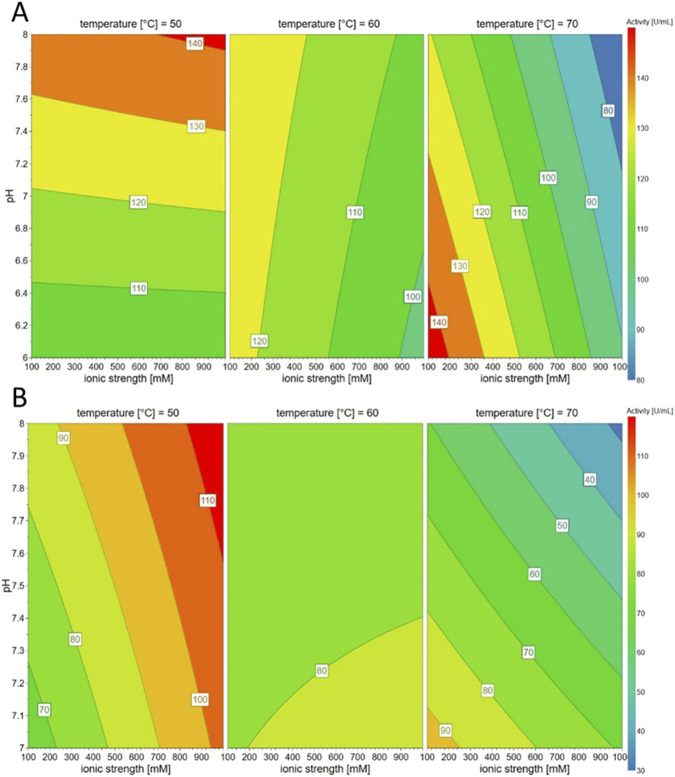
Hydrolysis of PBAT film and powder with the cutinase Kc_Cut from *K. chersonesos* **(A)** Ta concentration released over reaction time and **(B)** volumetric *p*-NPB activity during enzymatic reaction.

After the stability of the enzyme at the chosen temperature for the reaction time had been confirmed, hydrolysis of PBAT powder and film with Kc_Cut was studied. To confirm the preference for the KPO buffer as well during hydrolysis of polyesters, a preliminary trial was performed with PBAT powder which resulted in a Ta concentration of 12 ± 2 µM in KPO and 5 ± 0 µM in Tris-HCl buffer after 91 h. This indicated that the KPO buffer was preferred over the Tris-HCl buffer also during the hydrolysis process and therefore further experiments were performed in KPO buffer (100 mM, pH 8 and 50 °C). PBAT hydrolysis resulted in increasing concentrations of Ta over time (Figure 3A). The volumetric activity was tested during reaction and decreased over time in PBAT hydrolysis reactions which could also indicate irreversible binding of the enzyme to the polyester films which were not fully hydrolyzed after 72 h (Figure 3B). To examine this assumption, the remaining activity of 68.8 ± 17.2 U ml^-1^ was compared to the activity of enzyme at the same conditions without substrate after 72 h which resulted in 56.0 ± 0.3 U ml^-1^ (Supplementary Figure S8) and showed no significant difference. Therefore, the impact of substrate binding on decrease in volumetric activity can be neglected.

**FIGURE 4:**
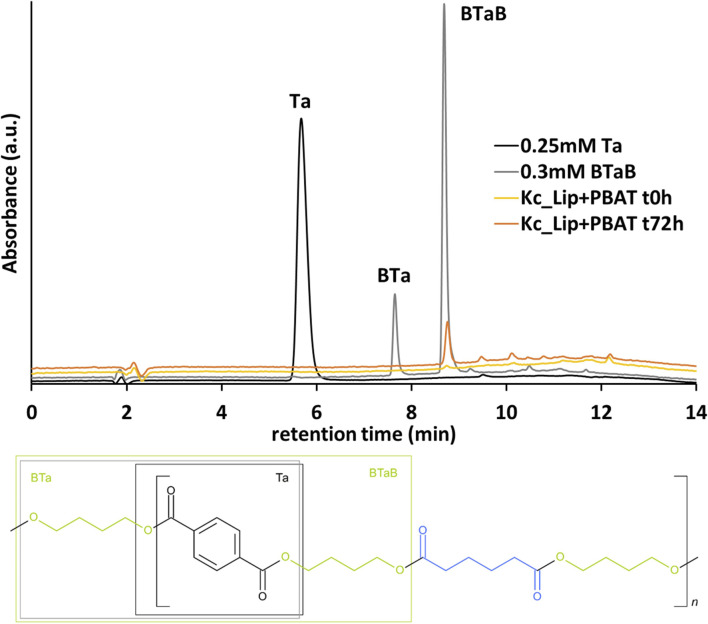
Release of BTaB by Kc_Lip. HPLC-UV signals detected after PBAT hydrolysis by Kc_Lip at t = 0 h and t = 72 h in comparison to Ta and BTaB standards with the molecular structure of PBAT shown in the bottom.

The hydrolysis reaction of PBAT with Kc_Lip was performed at 30 °C and did not result in detectable monomer (Ta) concentration. However, also the activity decreased by over 80% to 11 ± 0 U L^-1^. Though, using HPLC analysis the release of trimer (BTaB) was confirmed (Figure 4). This may indicate a cooperative reaction mechanism between Kc_Cut and Kc_Lip. Indeed, when both enzymes secreted by *K. chersonesos* are incubated with PBAT, in the first phase of hydrolysis up to 31% oligomers released which are later completely converted to monomers (Tesei et al., 2020). Hydrolysis of BTaB by Kc_Cut was tested and resulted in no residual BTaB concentrations from initial 0.3 mM which confirmed the hypothesis. Preference towards ester bonds between adipate and butanediol over terephthalate and butanediol has previously been described for a lipase from *Bacillus pumilus* and could also be assumed for Kc_Lip (Muroi et al., 2017). Very recently, cutinases from *Gordonia phtalatica* (Gph_Cut1) and *Gordonia paraffinivorans* (Gpa_Cut1 and Gpa_Cut2) revealed similar activities on PBAT film (Steiner et al., 2025). After 48 h of incubation at 50 °C and pH 8.0, the recombinant cutinases released 20.0 ± 2.8 µM (Gph_Cut1), 25.5 µM (Gpa_Cut1) and 5.6 µM (Gpa_Cut2) Ta.

## 4 Conclusion and outlook

This study reports the identification and functional characterization of two enzymes from the polyextremotolerant organism *K. chersonesos*. Kc_Cut and Kc_Lip were expressed in the eukaryotic organisms *K. phaffii* and *T. reesei*, respectively, whereby the expression levels could still be optimized. Both enzymes were able to hydrolyze PBAT but evaluation of optimal reaction conditions by using a DoE did not reveal extraordinary thermostability as could be expected for enzymes of this organism. The role of the isolated enzymes in PBAT hydrolysis is most likely cooperative, as the lipase released the trimer BTaB, which is to be expected for single enzyme reactions. Future studies should include testing enzyme activity on different polymers and further identification of the reaction mechanism. Therefore, experiments involving both enzymes (Kc_Cut and Kc_Lip) need to be performed for quantifying the cooperative action after optimizing the expression systems. The cooperative action could be assumed to be similar to the PETase and MHETase stepwise hydrolysis in *Ideonella sakaiensis*. The discovery of new enzymes should contribute to understanding the distribution of PBAT-hydrolyzing enzymes, as well as the adaptability, survival mechanisms and biotechnological potential of extremophilic organisms.

## Data Availability

The proteomics datasets generated during the current study are available in the ProteomeXchange Consortium (http://proteomecentral.proteomexchange.org) via the PRIDE partner repository with the dataset identifier PXD014026.
