# Supplementary material for: Life on the rocks: unexpected enzyme activity of the extremophilic black fungus Knufia chersonesos
Source: Front Bioeng Biotechnol. 2026 Jan 12;13:1720118. doi: 10.3389/fbioe.2025.1720118 (PMC12832766; doi:10.3389/fbioe.2025.1720118)
Supplement: Supplementary file 1 [file DataSheet1.docx]

Supplementary Material


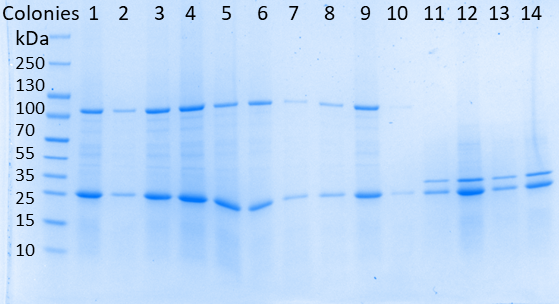


**Supplementary Figure S1.** SDS-PAGE gel of several transformed colonies expressing Kc_Cut to identify expression levels. Kc_Cut is visible at a molecular size close to 25 kDa which resembles the theoretical size of 22.36 kDa. The second visible protein represents the deglycosylase (Endo Hf in colonies 1-10 and Endo H in colonies 11-14).

1. Kc_Lip amino acid sequence

MYRKLAVISAFLATARAQAQQQRLMPQSDIWNSTYEVTQDRARGANLTDAELHDISVALNFERSNWATGSVADDEFYTLPSNASSASPGSVVKVQAYTNTSTYTLPPNTALSRIIFMTEDLNGTAVPASAYVLWPYLPRTQADGRYPLVTWGHGTSGGFAECGPSHIRNLWYQYSAPYALALAGYVVVAPDYRGLGINETANGKPIYHSYGAGQSAGIDLLYAAQAAQSAFPSISENFVVMGHSQGGNAAWGAAVRQAQSPSAGYLGTIAGSPTTNYTAIIEFYSGNPIIPPQLQLLWANALRGLHPSFNLSTILTNTGIARLNLASELGMCNSAVGMLLPSGSSTYVVQPDWLSVPELSSYLSLLDRGTQQEVAGPMLVLQGTLDPAVPEQITTAAVQSTCKLYPTSDIEYWLFANVTHVPVLYASQRLWLDWIAERFAAADNAEDSNGAGSDGSCSMKNFTSGPMPSENYQTELEYYLELATQGHQVAWSHPQFEK*

1. Kc_Lip nucleotide sequence

CTTAAG**ATG**TACCGAAAACTGGCCGTAATAAGCGCCTTCCTCGCGACCGCTCGAGCTCAGGCCCAACAGCAGCGCCTCATGCCCCAATCAGATATCTGGAACAGCACATACGAGGTCACGCAAGACCGAGCGAGGGGAGCAAACCTGACCGACGCTGAGCTGCATGACATTTCTGTTGCCTTGAACTTCGAAAGAAGTAACTGGGCAACGGGATCAGTCGCCGATGACGAGTTTTACACCCTGCCTAGCAACGCGTCCAGCGCAAGTCCTGGGTCGGTGGTCAAGGTCCAAGCTTATACGAACACATCCACTTACACGCTCCCTCCCAACACCGCCTTATCGCGCATCATCTTCATGACGGAGGACCTCAATGGCACGGCTGTTCCAGCCTCTGCTTACGTCCTCTGGCCGTATCTGCCGAGAACGCAAGCGGATGGTCGGTACCCCTTGGTCACCTGGGGCCACGGCACTTCAGGTGGTTTTGCCGAGTGCGGGCCTTCTCACATCCGAAATCTGTGGTATCAGTACTCTGCTCCCTACGCCCTGGCGCTTGCTGGCTACGTGGTTGTCGCTCCAGACTACAGGGGGCTCGGTATCAACGAGACGGCTAACGGCAAGCCCATCTACCACAGCTACGGCGCTGGTCAGAGTGCGGGCATCGACCTATTGTATGCTGCGCAGGCGGCGCAATCGGCCTTTCCCAGTATCTCCGAAAACTTCGTCGTCATGGGCCATTCACAAGGAGGAAACGCTGCCTGGGGTGCCGCCGTCCGCCAGGCCCAGTCCCCGTCCGCCGGATATCTCGGCACCATTGCCGGCAGCCCCACGACTAATTACACTGCCATCATTGAGTTCTACAGCGGCAACCCTATAATTCCGCCGCAGTTACAGCTGTTGTGGGCCAATGCCCTAAGGGGACTCCACCCGTCCTTCAACCTGTCGACGATCCTCACCAACACGGGGATTGCGCGGCTGAACCTCGCATCTGAGCTTGGCATGTGCAACTCGGCAGTCGGCATGCTCCTGCCATCTGGCTCTAGTACCTACGTCGTCCAACCTGACTGGTTGAGCGTACCAGAACTCTCCTCCTATCTGTCCCTCCTTGACCGCGGGACACAGCAGGAGGTTGCGGGACCCATGCTGGTGCTGCAGGGCACACTGGATCCGGCCGTGCCCGAGCAGATCACCACCGCAGCTGTGCAGTCGACCTGCAAGCTCTACCCAACCAGCGATATTGAGTACTGGCTCTTTGCCAATGTTACGCACGTCCCGGTGCTTTATGCATCACAGCGTTTGTGGCTGGACTGGATCGCCGAGCGCTTCGCCGCAGCCGACAACGCAGAAGACAGCAATGGCGCAGGCTCCGATGGTTCGTGTAGCATGAAGAACTTTACTTCGGGCCCGATGCCTAGCGAGAACTACCAGACCGAGCTCGAATACTACCTTGAACTGGCCACCCAGGGTCACCAGGTGGCGTGGTCGCATCCCCAGTTCGAGAAG**TAG**ACTAGT

**Supplementary Figure S2.** A.) Amino acid sequence of *Knufia chersonesos* secretory lipase Kc_Lip g1109.t1 carrying the N-terminal secretion signal peptide from *T. reesei* CBHI (orange) and the C-terminal StrepTag II (red) for expression in *T.reesei*. B.) Optimized nucleotide sequence of Kc_Lip. Bold: Start- and Stop codon of the Kc_Lip gene.


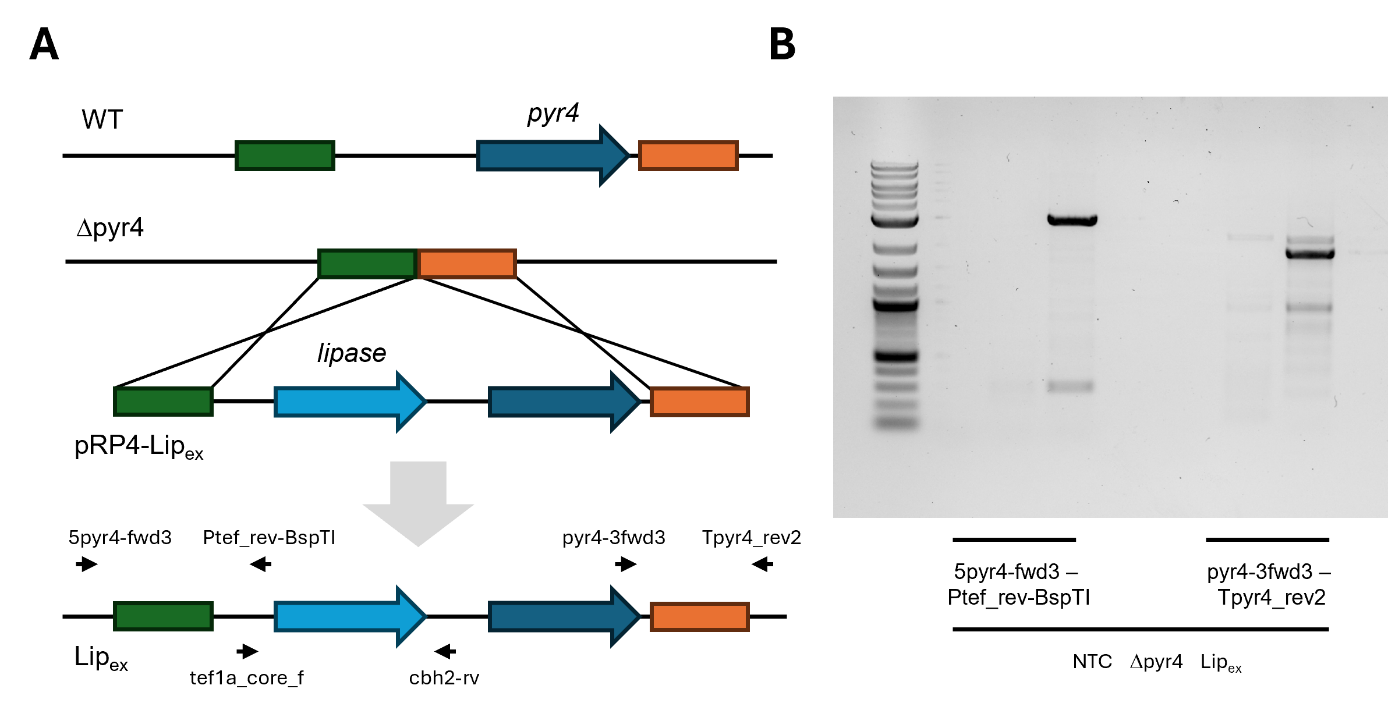


**Supplementary Figure S3**. Construction of the strain *T. reesei* Lip_ex._ A) The coding region for the Kc_Lip was put under the control of the *tef1* promoter and inserted into the *pyr4* locus of the recipient strain *T. reesei* QM6a Δpyr4 using a homologous insertion strategy as described previously (Derntl et al., 2015) B.) The insertion of the Kc_Lip expression cassette was verified by suitable PCR assays using the indicated primers and the chromosomal DNA of the used strains. NTC, no template control. The NEB 1kb DNA Ladder Plus was used as marker.

**Suppplementary Table 1**. Nucleotide primers used in this study

| **Primer name** | **Sequence (5’-3’)** |
| --- | --- |
| 5pyr4-fwd3 | CCAGACGGTGATTCACATATACG |
| Ptef_rev-BspTI | CTTAAGTGTGATGTAGCGTGAGAGCTG |
| pyr4-3fwd3 | TGCCTTTATCCACATGACGC |
| Tpyr4_rev2 | CAGGAAGCTCAGCGTCGAG |
| tef1a_core_f | TCGCCTTATTCTTCTTCCCTCTTCTG |
| cbh2-rv | CAAAGACTCCGCCAACC |


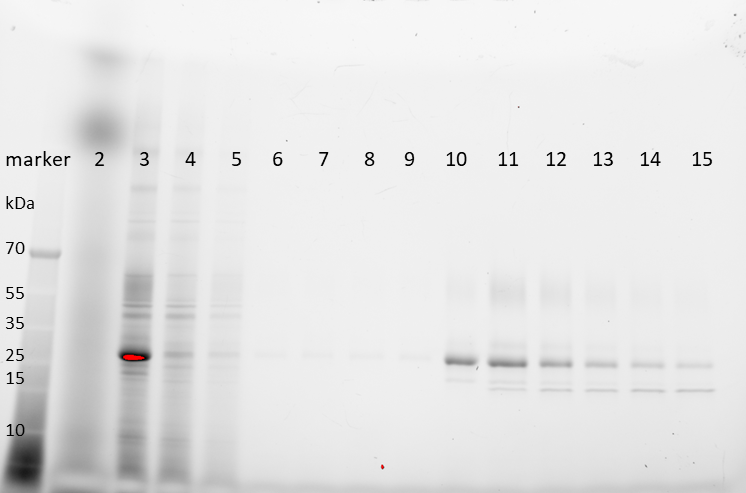


**Supplementary Figure S4.** SDS-PAGE of fractions during cutinase purification. Lane 1) Protein marker; Lane 2) permeate after concentration of cultivation supernatant; Lane 3) retentate after concentration of cultivation supernatant; Lanes 4-5) flowthrough; Lanes 6-15) elution fractions.

**Supplementary Table S2.** Molar extinction coefficients ($\varepsilon$) of *para*-nitrophenol

|  | KPO |  |  | Tris-HCl |  |  |
| --- | --- | --- | --- | --- | --- | --- |
| **mM** | **100** | **500** | **1000** | **100** | **500** | **1000** |
| pH |  |  |  |  |  |  |
| 6 | 1.49 | 1.72 | 1.13 |  |  |  |
| 7 | 8.36 | 6.36 | 5.87 | 10.48 | 14.21 | 15.37 |
| 8 | 17.51 | 15.51 | 16.19 | 18.98 | 18.76 | 19.27 |

Supplementary Table S3 Design of experiments for biochemical characterization of Kc_Cut in potassium phosphate buffer

| **Experiment No** | **Experiment name** | **Run order** | **Incl/Excl** | **Ionic strength**  **[mM]** | **pH** | **Temperature**  **[°C]** |
| --- | --- | --- | --- | --- | --- | --- |
| 1 | N1 | 21 | Incl | 100 | 6 | 50 |
| 2 | N2 | 22 | Incl | 500 | 6 | 50 |
| 3 | N3 | 23 | Incl | 1000 | 6 | 50 |
| 4 | N4 | 8 | Incl | 100 | 7 | 50 |
| 5 | N5 | 25 | Incl | 500 | 7 | 50 |
| 6 | N6 | 16 | Incl | 1000 | 7 | 50 |
| 7 | N7 | 29 | Incl | 100 | 8 | 50 |
| 8 | N8 | 1 | Incl | 500 | 8 | 50 |
| 9 | N9 | 14 | Incl | 1000 | 8 | 50 |
| 10 | N10 | 10 | Incl | 100 | 6 | 60 |
| 11 | N11 | 2 | Incl | 500 | 6 | 60 |
| 12 | N12 | 4 | Incl | 1000 | 6 | 60 |
| 13 | N13 | 27 | Incl | 100 | 7 | 60 |
| 14 | N14 | 9 | Incl | 500 | 7 | 60 |
| 15 | N15 | 18 | Incl | 1000 | 7 | 60 |
| 16 | N16 | 19 | Incl | 100 | 8 | 60 |
| 17 | N17 | 12 | Incl | 500 | 8 | 60 |
| 18 | N18 | 13 | Incl | 1000 | 8 | 60 |
| 19 | N19 | 30 | Incl | 100 | 6 | 70 |
| 20 | N20 | 15 | Incl | 500 | 6 | 70 |
| 21 | N21 | 20 | Incl | 1000 | 6 | 70 |
| 22 | N22 | 5 | Incl | 100 | 7 | 70 |
| 23 | N23 | 26 | Incl | 500 | 7 | 70 |
| 24 | N24 | 7 | Incl | 1000 | 7 | 70 |
| 25 | N25 | 3 | Incl | 100 | 8 | 70 |
| 26 | N26 | 11 | Incl | 500 | 8 | 70 |
| 27 | N27 | 6 | Incl | 1000 | 8 | 70 |
| 28 | N28 | 24 | Incl | 500 | 7 | 60 |
| 29 | N29 | 28 | Incl | 500 | 7 | 60 |
| 30 | N30 | 17 | Incl | 500 | 7 | 60 |

Supplementary Table S4 Design of experiments for biochemical characterization of Kc_Cut in Tris-HCl buffer

| **Experiment No** | **Experiment name** | **Run order** | **Incl/Excl** | **Ionic strength**  **[mM]** | **pH** | **Temperature**  **[°C]** |
| --- | --- | --- | --- | --- | --- | --- |
| 1 | N1 | 9 | Incl | 100 | 7 | 50 |
| 2 | N2 | 5 | Incl | 500 | 7 | 50 |
| 3 | N3 | 13 | Incl | 1000 | 7 | 50 |
| 4 | N4 | 16 | Incl | 100 | 8 | 50 |
| 5 | N5 | 20 | Incl | 500 | 8 | 50 |
| 6 | N6 | 10 | Incl | 1000 | 8 | 50 |
| 7 | N7 | 11 | Incl | 100 | 7 | 60 |
| 8 | N8 | 21 | Incl | 500 | 7 | 60 |
| 9 | N9 | 3 | Incl | 1000 | 7 | 60 |
| 10 | N10 | 17 | Incl | 100 | 8 | 60 |
| 11 | N11 | 14 | Incl | 500 | 8 | 60 |
| 12 | N12 | 19 | Incl | 1000 | 8 | 60 |
| 13 | N13 | 2 | Incl | 100 | 7 | 70 |
| 14 | N14 | 1 | Incl | 500 | 7 | 70 |
| 15 | N15 | 6 | Incl | 1000 | 7 | 70 |
| 16 | N16 | 8 | Incl | 100 | 8 | 70 |
| 17 | N17 | 12 | Incl | 500 | 8 | 70 |
| 18 | N18 | 4 | Incl | 1000 | 8 | 70 |
| 19 | N19 | 7 | Incl | 500 | 7 | 60 |
| 20 | N20 | 18 | Incl | 500 | 7 | 60 |
| 21 | N21 | 15 | Incl | 500 | 7 | 60 |


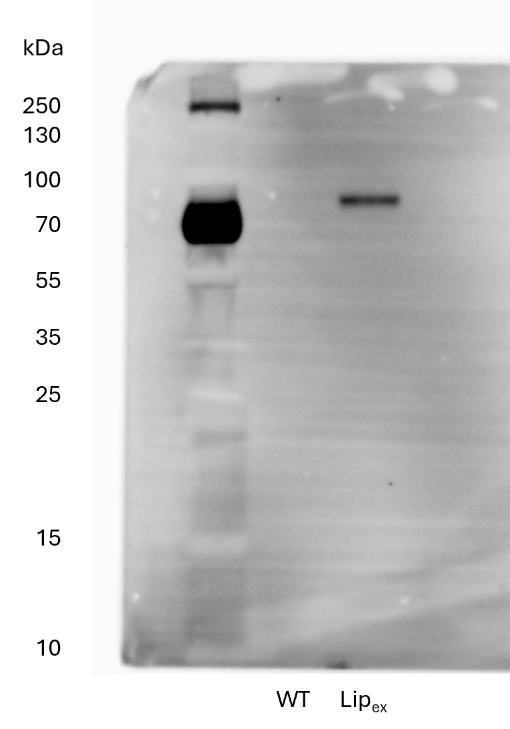


**Supplementary Figure S5**. Detection of the Strep-tagged lipase in the culture supernatant
Sample of the culture supernatant of the comparison strain QM6a Δmus53 (WT) and the lipase expression strain Lip_ex_ were separated on a discontinuous SDS-PAGE and blotted onto a PVDF membrane. Proteins with a Strep tag were detected using a polyclonal mouse antibody against the Strep tag, a goat anti mouse Poly-HRP antibody. The PageRuler Plus Prestained Protein Ladder (Thermo Scientific) was used as marker.


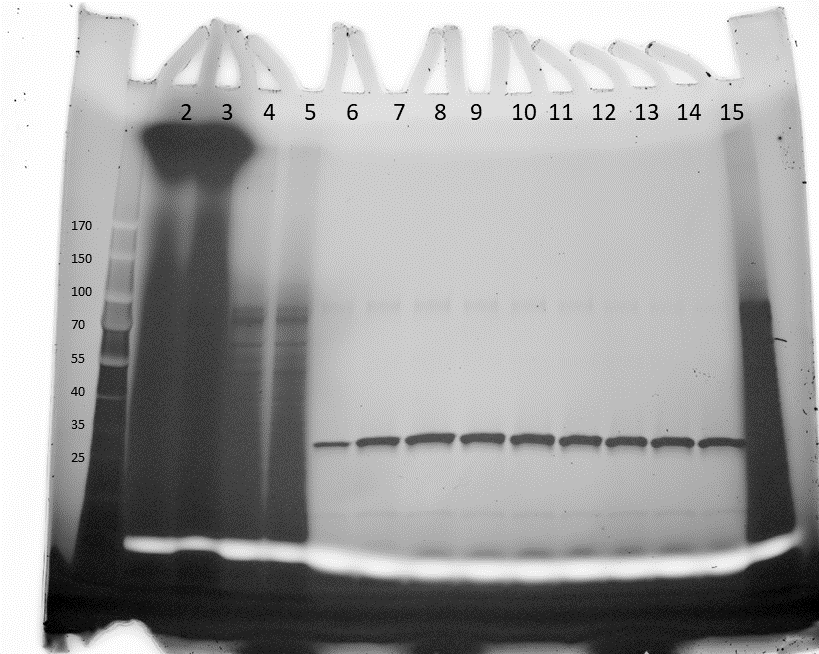


Supplementary Figure S6 SDS-PAGE of lipase purification expressed in *Trichoderma reesei*. Lane 2) filtration permeate; Lane 3) filtration retentate; Lanes 4-5) Äkta flowthrough; Lanes 6-14) elution peak fractions; Lane 15) filtration retentate after rebuffering.


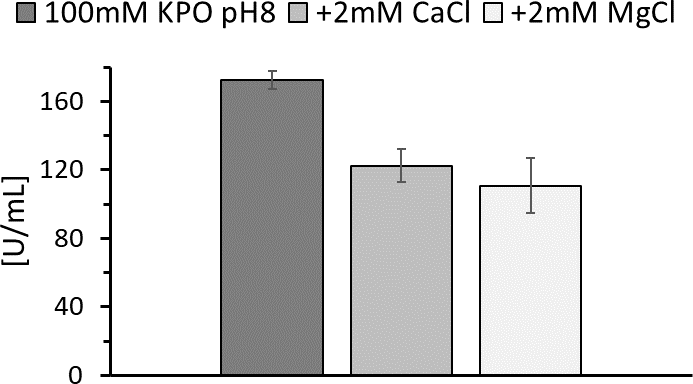


Figure S7. Impact of presence of 2 mM salts (CaCl and MgCl) on Kc__Cut activity measured by the *p*-NPB assay at 50°C.


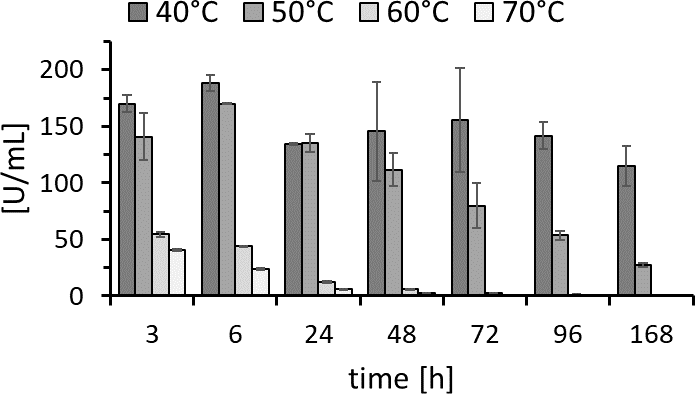


Supplementary Figure S8. Temperature stability determination of Kc_Cut through *p*-NPB activity assay at different temperatures for one week.
